# Probability of Medication Intensifications in Rheumatoid Arthritis Patients With Low Disease Activity Scores on Their Patient-Reported Outcomes: A Medical-Records Review Study

**DOI:** 10.1097/RHU.0000000000001883

**Published:** 2022-07-30

**Authors:** Bart Fabian Seppen, Simone J. Verkleij, Jimmy Wiegel, Marieke M. ter Wee, Michael T. Nurmohamed, Wouter H. Bos

**Affiliations:** From the ∗ Reade Rheumatology; † Department of Rheumatology, Amsterdam UMC; ‡ Department of Epidemiology and Data Science, Amsterdam UMC, Vrije Universiteit Amsterdam, Amsterdam, the Netherlands.

**Keywords:** patient-reported outcome, predictive value, rheumatoid arthritis, telemonitoring, treatment intensification

## Abstract

Patients with rheumatoid arthritis require frequent consultations to monitor disease activity and intensify medication when treatment targets are not met. However, because most patients are in remission during follow-up, it should be possible to reduce the number of consultations for them. Electronic patient-reported outcomes (ePROs) could be used to identify patients who meet their treatment goal and who could therefore be eligible to skip their visit.

**Objective:**

The aim of this study was to assess the probability that patients with low disease activity scores on their ePROs do not need a disease-modifying antirheumatic drug (DMARD) or steroid intensification in the first 2 weeks after completion of the ePROs.

**Methods:**

This medical-records review study compared results of ePROs answered during routine care with DMARD or steroid intensifications collected from anonymized electronic medical record at Reade. The primary outcome was the positive predictive value (PPV) of having a low disease activity score on an ePRO for not receiving a DMARD or steroid intensifications within 2 weeks. The 3 studied ePROs (and respective low disease activity outcome) were the Routine Assessment of Patient Index Data 3 (RAPID3) (score <2), Patient Acceptable Symptom State (PASS) (yes), and the flare question (no). The secondary aim of the study was to assess which combination of ePROs resulted in the best PPV for DMARD or steroid intensifications.

**Results:**

Of the 400 randomly selected records, ultimately 321 were included (302 unique patients). The PPV of a RAPID3 <2, being in PASS, and a negative answer on the flare question were, respectively, 99%, 95%, and 83% to not receive a DMARD or steroid intensification within 2 weeks. The combination of a RAPID3 <2 and a negative flare question resulted in a PPV of 100%; this combination was present in 29% (93/321) of the total study population.

**Conclusion:**

The RAPID3, PASS, and flare question have a high diagnostic accuracy to identify individuals who will not receive a DMARD or steroid intensification in the following 2 weeks. The combination of the RAPID3 and flare question yielded the best combination of diagnostic accuracy and highest percentage of patients who could be eligible to skip a visit. These results suggest that accurate identification of patients who meet their treatment goal with ePROs is possible.

The disease activity of patients with rheumatoid arthritis (RA) is frequently monitored at the outpatient clinic following treat-to-target principles, with the goal to achieve remission or low disease activity.^1,2^ Traditionally, patients are monitored 2 to 4 times a year with composite measures such as the Disease Activity Score 28 (DAS28) or Clinical Disease Activity Index during preplanned visits, and therapy is intensified when treatment goals are not met.^1,2^ Most patients, however, meet their treatment goal. Therefore, the current system results in a vast amount of (potentially) unnecessary consultations. These consultations consume so much health care costs and time that the current system will not be sustainable in the future: the projected number of rheumatologists needed to sustain the system is twice the number of available rheumatologists by 2030.^3,4^ Therefore, ways should be sought to lower the number of outpatient clinic visits for patients in remission or low disease activity.

If we could identify patients who meet their treatment target (low disease activity or remission) before consultations, we could let them skip their visit. For this to work, we would need to (1) assess disease activity without a consultation and (2) be confident that patients with low disease activity assessments truly are in remission. Disease activity assessment with electronic patient-reported outcomes (ePROs) could meet both requirements, as ePROs are scored independently by patients and tend to overestimate disease activity. This overestimation should lead to a low number of patients who are falsely classified in remission.

Several ePROs such as the Routine Assessment of Patient Index Data 3 (RAPID3),^5^ the Patient Acceptable Symptom State (PASS),^6^ and the flare question “Are you having a flare of your RA at this time?” might be suitable to identify patients who are in low disease activity.^7^ This is because patients who are in RAPID3 remission or low disease activity are in more than 90% of the cases also in DAS28 low disease activity or remission.^8,9^ Furthermore, patients have a relatively low pain threshold (pain visual analog score of 36/100) to classify their symptoms as not acceptable in the PASS.^6^ This makes it likely that patients who classify their symptoms as acceptable in the PASS truly have low disease activity. And lastly, patients in remission have a high agreement (*κ* >0.73) with their physician regarding their nonflare status.^6–8^ However, whether identification of patients who meet their treatment target is possible with these ePROs is unknown.

So far, research has focused on the relationship between ePROs and clinical composite measures. In practice, however, the decision to intensify treatment when arthritis is active is more complex than not meeting a DAS28 or Clinical Disease Activity Index threshold.^10,11^ Therefore, the relationship between the ePROs and actual disease-modifying antirheumatic drug (DMARD) or steroid intensification (rather than doctor-measured disease outcomes) will be key in the assessment whether accurate identification of patients in low disease activity is possible. Very little is known about the relationship of the RAPID3, PASS, and flare question with treatment intensifications. This study will evaluate the relationship between the results of 3 ePROs (RAPID3, PASS, and flare question) and receiving a DMARD and/or steroid intensification, as a preparatory step toward identifying patients in remission or low disease activity before outpatient clinics to let them skip their visits. We hypothesize that patients who report to be in remission or low disease activity according to their RAPID3 score, PASS, or flare question answer will receive very few DMARD or steroid intensifications.

## METHODS

This was a medical records study performed at Reade, a secondary care facility for rehabilitation and rheumatology in Amsterdam, the Netherlands. All patients in routine care who are scheduled for a telephone call or outpatient clinic visit with a rheumatologist or a rheumatology nurse received an invitation by mail to complete the RAPID3, PASS, and flare question in an online portal before their consultation. The results of the ePROs and the corresponding patients' demographics (sex, age, disease duration, and current use of medication for RA) of each patient were extracted from the electronic medical records. Records (results of the ePROs) were included if they were completed between March 1, 2020, and January 15, 2021, and they regarded adult (aged ≥18 years) RA patients. Records were excluded if they regarded a patient with a disease duration less than 1 year (newly diagnosed patients were not considered to be eligible to skip their visits) or if no consultation took place within 2 weeks of the completed ePROs (to minimize the risk of changes in disease activity between completing ePROs and the consultation). Records that were completed within a 3-month follow-up time frame of an already included ePRO record of the same patient were also excluded. All eligible records were ranked using a random-number generator in SPSS (SPSS Inc, Chicago, IL), and the first 400 ePRO records were included in this study.

Disease-modifying antirheumatic drug or steroid intensifications were looked up in anonymized patient files extracted from the electronic medical records of Reade by one researcher (S.J.V.). Cases were discussed with a second researcher (B.F.S.) in case of doubt. Both researchers were unaware of the patients' ePRO results during this process. Disease-modifying antirheumatic drug or steroid adjustments were recorded as “direct” (within 2 weeks) or “during follow-up” (within 3 months). The 2-week time frame was chosen as patients who are initially called and suspected to be flaring are seen within 2 weeks at the outpatient clinic for an in-person follow-up.

### Ethics

The procedures were performed according to legislations of the Medical Research Involving Human Subjects Act and Dutch ethical standards for research involving humans. Because this was a study with anonymized medical files, no approval from the ethics committee was needed.

### Outcome Measures

The primary outcome of this study was the positive predictive value (PPV) of a low RAPID3 score, being in PASS, or not reporting a flare for direct DMARD or steroid intensifications. This result can be interpreted as the probability that a patient with a low RAPID3 score, who is in PASS, or who reports not to be flaring does not receive a DMARD or steroid intensification.

The RAPID3 categorizes patients into remission (0–1.0), low (1.1–2.0), moderate (2.1–4.0), or high disease activity (>4). These categories were dichotomized into a “low” (≤2) and “high” (>2) RAPID3 score.^5^

The PASS is a single yes or no question: “Considering all different ways RA is affecting you, if you were to stay in this state for the next 3 months, do you consider your current state satisfactory?” Patients who answered “yes,” indicating they were satisfied with the current status of their RA, were defined as patients “in PASS.” Patients who answered “no” were defined as patients “not in PASS.”^6^

The *flare question* is a single yes or no question from the RA Flare Core Domain set.^7^ Patients were asked if they experienced a flare: “Are you having a flare of your RA at this time?”

Disease-modifying antirheumatic drug or steroid intensifications were defined as an increase in dosage, adding or switching prednisone (oral and intramuscular), conventional synthetic, biologic, or targeted synthetic DMARDs. In addition, intentions to intensify DMARDs were recorded when they were formulated by a rheumatologist, but the intensification was not executed because of refusal by a patient. Both actual intensifications and intentions to intensify were considered as DMARD or steroid intensifications in the analysis.

Medication reductions were defined as a decrease in dosage or stopping medication. If medication was temporarily halted in case of an infection or before an operation, this was not registered as a reduction. Tapering of short-term high-dose prednisone was also not counted as a reduction.

### Statistical Analysis

In case of normally distributed data, data were summarized using means and SD. Dichotomous data were summarized using frequencies and percentages. In case of not normally distributed data, data were summarized using medians and interquartile range. For these dichotomous data, we used a χ^2^ test, or if one of the cells had a frequency <5, a Fisher exact test. The *α* was set at .05. No sample size calculation was performed as no accurate method exists to calculate the power for this type of study.^12^ Following the Bland-Altman method, a 100-subject sample usually considered a good sample size to evaluate agreement. Therefore, we included a 100-subject sample per variable (PASS, flare, and RAPID3) to assess the agreement with DMARD intensifications. We chose to assess an extra 100 (total 400 records) to account for excluded records.

Test measurement properties (the sensitivity, specificity, PPV, and negative predictive value [NPV]) with 95% confidence intervals (95% CIs) were calculated. This was done for both direct DMARD or steroid intensifications and during follow-up. A RAPID3 ≤2, being in PASS, and reporting not to be flaring were considered as a positive diagnostic test result; henceforth, not receiving a DMARD or steroid intensification was considered as the criterion standard. Electronic patient-reported outcomes with a high specificity can be used to identify the presence of a “condition,” in this case low disease activity. The PPV is the chance of having this condition when the ePROs show a positive test. To test the accuracy of the ePROs, receiver operating characteristic curves were calculated, yielding area under the curve (AUC) values.

As secondary outcomes, all combinations of the ePROs were evaluated with a cross-tabulation to assess which combination had the best PPV and AUC. In addition, for each ePRO and the best combination of ePROs, the intensification-free survival was displayed in a Kaplan-Meier curve. All statistical analyses were performed in SPSS version 23 (SPSS Inc).

## RESULTS

### Patient Selection

Between the March 1, 2020, and the January 15, 2021, the RAPID3, PASS, and flare question were completed 3688 times. Of the 400 randomly selected ePRO records, 79 were excluded for the following reasons: an inconclusive RA diagnosis, despite the RA treatment code (n = 7); disease duration <1 year (n = 48); no consultation within 2 weeks of the completed ePROs (n = 21); and ePRO records within 3 months of a previously completed ePRO record of the same patient (n = 3). The 321 records that were included were completed by 302 patients (19 patients with 2 entries); characteristics of the study population are shown in Table 1. In the study population 20% (65/321) of all patients received at least 1 DMARD or steroid intensification in the first 2 weeks after their first consultation (including intensification during their first consultation). In total, 30% (96/321) of the patients had a low RAPID3 score, 60% (191/321) was in PASS, and 71% (229/321) reported not to be flaring.

**TABLE 1:** Patient Characteristics (n = 302)

Total Measurements	321	
Total patients*	302	
Female, n (%)	231	(77)
Age, years	60	(12)
Disease duration, median (interquartile range), y	10	(4–16)
B-DMARDs or ts-DMARDs, n (%)	134	(44)
RAPID3	3.7	(2.3)

*Primary (in time) characteristics of patients with 2 records are presented. Data are presented mean (SD) unless otherwise indicated.

B-DMARDs, biological DMARDs; ts-DMARDs, targeted synthetic DMARDS.

### Primary Outcome

The percentage of patients who received a direct DMARD or steroid intensification or during the follow-up was significantly less in patients with a low RAPID3 compared with patients with a high RAPID3 (direct: 1% vs 28%, *p* < 0.001; during follow-up: 5% vs 39%, *p* < 0.001), in patients in PASS compared with patients not in PASS (direct: 5% vs 42%, *p* < 0.001; during follow-up: 12% vs 54%, *p* < 0.001), and in patients who reported not to be flaring compared with patients who reported to be flaring (direct: 7% vs 52%, *p* < 0.001; during follow-up: 15% vs 63%, *p* < 0.001). The respective sensitivity, specificity, NPV, and PPV of each ePRO are displayed in Table 2.

**TABLE 2:** Predictive Values of ePROs for DMARD or Steroid Intensifications

ePROs	PPV	Specificity	NPV	Sensitivity
Direct (<2 wk)				
RAPID3	99	99	28	37
PASS	95	85	42	71
FLARE	93	74	52	83
RAPID3 + flare	100	100	36	29
Follow-up (<3 mo)				
RAPID3	95	95	39	40
PASS	88	75	54	74
FLARE	85	62	63	85
RAPID3 + flare	96	96	39	39

All numbers are percentages.

### Secondary Outcome

The combination of the RAPID3 and the flare question led to a PPV and specificity of 100%; this combination was present in 29% (93/321) of the study population; adding the PASS to this combination did not improve the test characteristics (lower AUC 0.682 vs 0.672) and reduced the size of the group that would have been eligible to skip their visit. The results of the 2 × 2 cross-tabulations of all combinations can be found in Appendix 1, http://links.lww.com/RHU/A486.

The DMARD or steroid intensification-free survival during the 90 days of follow-up is displayed in Figure. In the group of patients with a low RAPID3 and that reported not to be flaring, the first DMARD or steroid intensification was prescribed after 40 days. The intensification-free survival curves of the separate RAPID3 and RAPID3 combined with the flare question were very similar.

**FIGURE:**
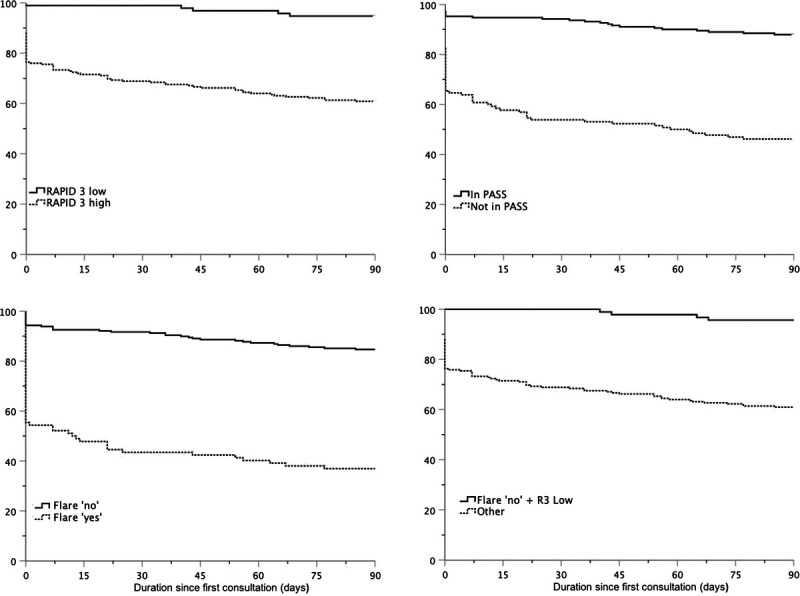
DMARD or steroid intensification-free survival (% of patients). Disease-modifying antirheumatic drug or steroid intensification-free survival in patients with a low or high RAPID3 score (top left), patients (not) in PASS (top right), patients who were (not) flaring (bottom left) and patients with favorable outcomes on the RAPID3 and flare question (low RAPID3 and flare ‘no’) and patients with at least one unfavorable outcome (high RAPID3 and/or flare ‘yes’) (bottom right). All patients were followed for the complete 3 months of follow-up.

Disease-modifying antirheumatic drugs were reduced within 2 weeks in 8% (24/321) of the total study population; 6 additional reductions were registered in the remainder of the follow-up period. Respectively, 12 of 24 (50%) and 13 of 30 (43%) of these reductions were registered in the low RAPID3 and “no” flare group.

## DISCUSSION

To the best of our knowledge, this was the first study in patients with RA that investigated the predictive value of the RAPID3, PASS, and flare question for DMARD or steroid intensifications. Independently, a low RAPID3 had the best PPV (99%) for not receiving a direct DMARD or steroid intensification, but the PPVs of the PASS (95%) and flare question (93%) were also high. A low RAPID3 combined with not reporting a flare further increased the PPV to 100% and remained high during the 3-month follow-up (96%). This indicates that if patients with the combination of no (self-reported) flare and a low RAPID3 were to skip their visits, no patients who would need treatment intensification would be missed in the first 2 weeks.

Our results show that approximately one-third of the RA population has a low RAPID3 score and does not report a flare; these patients may be eligible to skip their visit on the basis of their ePRO results. Previously, Boone et al.^13^ indicated that monitoring disease activity with the RAPID3 alone captured changes in objective inflammatory signs insufficiently. This premise is supported by the low correlation of high RAPID3 scores with the DAS28. Nevertheless, our results suggest that the RAPID3 can be of value to identify stable patients, because patients who report a low RAPID3 score truly are in a low disease activity/remission state according to their DAS. This is demonstrated by the high predictive value of a low RAPID3 for low disease (according to the DAS28) by Wiegel et al.^8^ and Boone et al.^9^ and the low number of DMARD or steroid intensifications these patients receive in our study. Therefore, the RAPID3 could be used to identify patients in remission, especially when combined with the flare question. The combined results of the RAPID3 and the flare question demonstrated perfect (100%) results (in terms of PPV) within 2 weeks and excellent (96%) results after 3 months. This was further strengthened by the intensification-free survival curve that illustrated that in the first 40 days no DMARD or steroid intensifications would have been missed.

The sensitivity and NPV of the ePROs (and especially the RAPID3) were low, which indicates that many patients with high disease activity scores on their ePROs did not receive DMARD or steroid intensifications. This may be due to (1) the discrepancy between the patient and doctor assessed disease activity or (2) a failure to treat to target.^14,15^ As a result of the low sensitivity and NPV, there are still many patients who may be eligible to skip their visit, which have not been identified. There may be ePROs with a higher sensitivity and NPV that could identify a bigger proportion of patients who could be eligible to skip their visit. However, the priority is to not miss any patients who need a DMARD or steroid intensification. Therefore, the likely consequence for any PROM with a high specificity and PPV is that some patients who will not receive a DMARD or steroid intensification will also be called up. This could also have a beneficial effect, as the patients who have indicated some form of disease activity on their ePROs will be able to discuss it (and prevent further deterioration).

### Implications

Based on our data, it could be argued that the RAPID3 on its own misses only very few DMARD or steroid intensifications. However, we suggest combining the RAPID3 with the flare question. The flare question takes only a few extra seconds to answer, and combining it with the RAPID3 slightly increased the PPV without reducing the group size of patients. If these results are validated prospectively, ePROs could be used to allocate outpatient clinics visits according to need. The potential of this strategy is also suggested by the results of the SEMORA study that combined patient-initiated follow-up with telemonitoring with ePROs.^16^ In addition, identifying stable patients could be used in other strategies to reduce unnecessary visits, lower health care costs, and reduce manpower needed per patient. For instance, aside from letting patients skip consultations completely, an alternative strategy would be to replace the physical consultation with a rheumatologist with a nurse consultation by phone. This would decrease the workload of rheumatologists and has been shown to be just as good.^17–19^

### Generalizability and Limitations

The characteristics of this study population were comparable to characteristics of RA populations in Western countries.^20^ Because of the COVID-19 pandemic, there were more consultations by phone than usual. We do not believe this has significantly influenced our results, as the number of DMARD or steroid intensifications was in line with numbers reported by Saraux et al.^17^ (20%) and Dougados et al.^14^ (15%). This study has several limitations. First, the need for a consultation was defined as receiving a DMARD or steroid intensification. However, there may be other reasons for having to consult a rheumatologist, such as monitoring adverse events or reducing medication (dosage). However, these nonprescribing medical activities such as monitoring adverse events could also be performed by rheumatology nurses. Based on these data, 50% of the reductions would take place in the low RAPID3 and no-flare group. Therefore, it may be possible to actively screen this group for the possibility to reduce medication (dosage) with an additional question. In a prospective study, medication reductions should be a point of consideration. In addition, despite not receiving a DMARD intensification, disease activity might still be moderate or high. This might be considered a limitation, although previous studies have shown that the agreement of PROs with the DAS28 in the low disease activity categories is also high.^8,9^ Second, rheumatologists were not blinded from the ePRO records, which may have led to more treatment intensification in patients with undesirable ePRO results. However, this resembles the routine clinical practice in which the intervention would also be implemented and was therefore not considered as an issue. Third, identification of stable patients can only work if patients complete their ePROs. Our results are therefore applicable only to patients who complete their ePROs. In our setting, approximately half of the ePRO questionnaires are completed. Persuasive principles and optimization of the ePRO process could improve the completion rates.^21^

## CONCLUSION

The RAPID3, PASS, and flare question have a high diagnostic accuracy to identify patients who will not receive a DMARD or steroid intensification in the next 2 weeks. With the combination of a low RAPID3 and flare question, none of the patients received a DMARD or steroid intensification in the following 2 weeks. These results suggest that accurate identification of patients who meet their treatment goal with ePROs is possible.
